# “Goodbye … Through a Glass Door”: Emotional Experiences of Working in COVID-19 Acute Care Hospital Environments

**DOI:** 10.1177/0844562120982420

**Published:** 2021-03

**Authors:** Jennifer Lapum, Megan Nguyen, Suzanne Fredericks, Sannie Lai, Julie McShane

**Affiliations:** 1 Daphne Cockwell School of Nursing, Ryerson University, Toronto, ON, Canada; 2 Dalla Lana School of Public Health, University of Toronto, Toronto, ON, Canada; 3 Toronto General Hospital, University Health Network, Toronto, ON, Canada

**Keywords:** COVID-19, nursing, emotions, pandemic, Qualitative approaches, Narrative

## Abstract

**Background:**

The severity of the COVID-19 health crisis has placed acute care nurses in dire work environments in which they have had to deal with uncertainty, loss, and death on a constant basis. It is necessary to gain a better understanding of nurses’ experiences to develop interventions supportive of their emotional well-being.

**Purpose:**

The purpose of this study is to explore how nurses are emotionally affected working in COVID-19 acute care hospital environments. The research question is: What is the emotional experience of nurses working in COVID-19 acute care hospital environments?

**Methods:**

We employed a narrative methodology that focused on participants’ stories. Twenty registered nurses, who worked in six hospitals in the Greater Toronto Area in Canada, participated in interviews. A narrative analysis was conducted with a focus on content and form of stories.

**Results:**

We identified three themes about working in COVID-19 acute care hospital environments: the emotional experience, the agency of emotions, and how emotions shape nursing and practice.

**Conclusion:**

In moving forth with pandemic preparations, healthcare leaders and governments need to make sure that a nurse’s sacrifice is not all-encompassing. Supporting nurses’ emotional well-being and resilience is necessary to counterbalance the loss and trauma nurses go through.

## Background and purpose

The selfless sacrifice of nurses became the public discourse dominating headlines during the COVID-19 pandemic in 2020 (El-Masri & Roux, 2020; Freer, 2020). Nurses were applauded for their commitment to care for patients when society was generally alarmed by the nature of the pandemic, and sheltered in their homes. And yet, this storyline of sacrifice acts to divert attention away from the actual emotions that nurses are feeling (Freer, 2020) – emotions that can be dynamic, complex, and distressing. For decades, scholars have questioned how emotions can galvanize nurses to sacrifice, but also how this sacrifice can lead to emotional distress (Pask, 2005). Although the nurse’s sacrifice comes with the reward of helping others, we cannot discount that a sacrifice would not be a sacrifice without the potential for risk or loss.

The sacrifice for nurses globally has been sobering, and involved many becoming infected in the workplace with hundreds dying (Freer, 2020) and some dying by suicide (Rahman & Plummer, 2020). Although death may be the ultimate sacrifice, preliminary studies have reported that nurses experience emotional and psychological distress, insomnia, and an all-consuming fear that they may become infected and infect their families (Labrague & de los Santos, 2020; Lai et al., 2020), particularly during a time when there are many unknowns. With the severity of this crisis and the constant exposure to uncertainty, loss, and death, there are concerns for the vicarious trauma that nurses may experience (Reger et al., 2020) and their overall well-being in terms of distress, anxiety, and exhaustion (Jackson et al., 2020).

Nurses working in COVID-19 acute care hospital environments are particularly vulnerable considering that the environment is fraught with physically and emotionally demanding work complicated by the dynamic nature of COVID-19 in which the focus of care can shift rapidly from curative to palliative. As frontline providers, they spend sustained periods of time caring directly for patients and families, and working tirelessly to equip themselves with the knowledge about transmission, symptoms, diagnosis, and treatment of this novel virus. At the same time, they are dealing with many other issues involving compromised work conditions (Thorne, 2020), increased workload with long hours, inadequate personal protective equipment (PPE) (Iheduru-Anderson, 2020; Jackson et al., 2020), and a shifting knowledge base concerning COVID-19 transmission and virulence which has required them to engage in expanding their knowledge base and skill set (Stokes-Parish et al., 2020).

Prior to COVID-19, acute care environments were sometimes described as traumatic for nurses affecting their well-being (Allen & Palk, 2018) including their emotional health. Existing literature refers to emotions as feelings that shape the way we perceive situations, events, and other people (Zadra & Clore, 2011). As such, emotions are not neutral, but rather influence cognitive, physiological, behavioural, and psychological responses (Hayward & Tuckey, 2011). The demands and unknowns of COVID-19 may impact nurses’ emotional health by increasing the trauma they experience. Unmanaged negative emotions can have serious consequences on nurses’ well-being and their professional practice (Delgado et al., 2017). Because of their dynamic nature, emotions must be managed in proactive ways so that we adapt and protect ourselves from maladaptive emotions and associated responses (Hayward & Tuckey, 2011). This is important considering the emotional impact that traumatic work environments can have on nurses, particularly the potential impact of COVID-19 acute care environments.

The impact of not understanding nurses’ emotional experiences and not caring for these valuable frontline workers is multi-fold. Previous research has indicated that stressful work environments can lead to decrease in nurses’ quality of life, increase in sick time, decrease in the quality of care delivered (Sarafis et al., 2016), emotional exhaustion and burnout (Canadas-De la Fuente et al., 2015), and even decisions to leave the profession (Halter et al., 2017). We owe it to nurses to better understand their emotional experiences in order to develop supportive interventions during pandemics such as COVID-19. The purpose of this study is to explore how nurses are emotionally affected working in COVID-19 acute care hospital environments. The research question is: What are the emotional experiences of nurses working in COVID-19 acute care hospital environments?

## Methods and procedures

We employed a narrative methodology informed by Lieblich et al.’s (1998) approach focusing on storied components of experience. From this perspective, emotions are considered fundamentally “narrative in nature” (Kleres, 2010, p. 188) in that the configuration of these storied components are what constitute emotional experience. As such, our approach to analysis focused on how these narrative components configured to tell a story about nurses’ emotional experiences. In addition, we dissected the linguistic and paralinguistic features of the narratives, paying close attention to direct and indirect expressions of emotion (Lieblich et al., 1998).

A non-probability sampling method was used to recruit nurses who met the study inclusion criteria (Guest et al., 2006), which included registered nurses working in acute care hospital environments located in the Greater Toronto Area and working on units caring for COVID-19+ patients. Nurses were recruited from select hospital unit listservs as well social media. Interviews were conducted via Zoom which provided audio and video capacity based on participants’ preference. A semi-structured interview guide was used with questions such as: Can you tell me about how you are emotionally affected by working in a COVID-19 acute care hospital environment? How did these emotions feel? Interviews were transcribed with a group approach to analysis using Lieblich et al.’s (1998) narrative approach which focused on categorical content (e.g., what participants’ say) and form (how participants’ say it) of stories. The narrative coding structure was developed based on dialogical and iterative processes in which it was continuously adapted until the research team agreed that it comprehensively captured the data. Ethics approval was received from the hospital research ethics board (REB) where recruitment began as well as the university REB of the principal investigators.

## Results

Twenty nurses, including front-line providers and nurse leaders from six institutions, participated in the interviews that were about one hour. We use plural pronouns in referring to nurses (ie. they/them) and also do not define the specific nurse leadership positions in order to protect identity. In this section, we discuss each theme that emerged including: the emotional experience, the agency of emotions, and how emotions shape nursing and practice.

### The emotional experience

The emotional experience consisted of complex emotions that were deeply enmeshed with one another. The emotional experience included fear and apprehension, uncertainty, frustration and anger, helplessness and sadness, and resilience.

#### Fear and apprehension

Fear and apprehension were interwoven throughout nurses’ stories and related to a sense of impending danger and fear related to transmission. One nurse noted that it was “like scaling up a mountain you didn't know. No one wanted to go up. There was a lot of fear.” Others were “scared” because of seeing what happened in “Wuhan” and “New York … there were a lot of unknowns. … It can happen to us.” There was a lot of fear and apprehension “because of how contagious it was and the rates in which the cases were rising, just scared.” Fears also circulated around PPE: “On TV, you see how all these COVID centers, they're all in hazmats. All we had was just PPE … makes you think about whether this stuff is really protecting you.” Working on a COVID unit, one nurse described it as “stressful knowing that all of the people you're going to be taking care of have this virus that so many people are scared of.” In being a frontline worker, nurses were “scared” that they would contract it, as one nurse recalled “I'm gonna get it tonight. There's not enough masks, there's not enough PPE.” It was common for nurses to be fearful of transmitting it to their family. One nurse explained speaking to their family about the process of arriving home: “Unlock the door, get away from the door and I just go straight to the bathroom.” The “fear” of transmitting the virus to their family was prevalent and caused many nurses to make “sacrifices.” In this excerpt, a nurse describes the process of picking up their child after work:

My [young] daughter was trained to, don't come near me, don't hug me, wait until we get home, I'll go straight to the washroom, put everything in the washroom shower and then I'll come out, like, okay, I'm ready for the hug now.

One nurse recalled: “my kids” asked “if I was going to die … we [nurses] were all trying to get our wills in order just in case.” They were explicit about “the fear … either you or someone you love is going to pass away.” A nurse leader described feeling “scared … to fail my staff … not be able to guide them through this pandemic.” They elaborated on the fear that was felt at an organizational level:

We were tracking the numbers and we were seeing what happened in Italy, so we're freaking out … how are we going to do beds? How do we staff? How are we going to get ventilators? It's very scary to not know the right answer and the right way of doing things. There wasn't a manual.

Nurses’ experiences of fear were also closely entwined with feelings of uncertainty.

#### Uncertainty

Uncertainty permeated nurses’ stories in terms of worry and feelings of not knowing. Nurses referred to “uncertainty … we didn't know a lot about COVID at the time.” Another nurse described working in the environment as “going into the fire … because of the times of COVID and the severity of symptoms, you are going in with uncertainty.” They further elaborated on how the idea of the fire was agitated by the constantly changing information: “Every day policies and practices are being changed … it was like a fire of uncertainty and the fire of fear, not knowing what we're going to see that day.” One nurse commented that “one minute surgical mask and [then] it’s an N-95 … don't know who to believe or how to protect yourself, and protect your family.” The impact of uncertainty was that it “takes away my empowerment … adds to the anxiety and the stress level I have.” Another nurse commented: “it was hard to navigate … in my role when they’re coming towards you for leadership and guidance … for me it’s something new as well … kind of navigating new waters.” The narratives of uncertainty as well as fear also involved storied elements of frustration and anger.

#### Frustration and anger

Nurses experienced frustration and anger, which involved feeling upset and aggravated because of unfair situations: “my whole world basically just changed … everything was very unfair.” Many nurses were emotionally affected and upset because of the restricted visiting hours: “I felt very angry a lot of time … I felt it wasn't enough for family members.” Another nurse highlighted the frustration associated with the complexity of contact tracing referring to a patient who became infected with COVID in the hospital: “they suddenly deteriorated … very difficult to track sources … they swabbed everybody, and we had four people who tested positive and yet we don't know what the source was. So that was frustrating.” Nurses also expressed frustration and anger because they observed that hospitals and governments were not prepared: “they didn’t do what they said they were going to do after SARS which was, be ready for something … it’s like they’ve learned nothing.” They explained that nurses were being asked to “reuse our N-95” because there was a “lack of PPE.” This practice was upsetting because it “goes against everything we were taught about infection control. It makes us all feel angry because we've been let down. And we feel tense … if you're recycling all of this PPE, are you really protected?” As one nurse described, it felt like you were “a sacrificial lamb.” The anger and frustration was palpable in nurses’ stories: “I'm so fed up … going into the unit and risking my life and risking the lives of my kids and my husband, the anger that I feel is real and it's exhausting.” Oftentimes, the situations that led to anger and frustration also involved feelings of helplessness and sadness.

#### Helplessness and sadness

Nurses expressed feelings of helplessness and sadness when unable to affect a situation. One nurse felt helpless watching another nurse caring for a deteriorating patient, explaining that code teams need to don PPE and prepare medications before entering:

The patient’s oxygen was going lower. … you can hear her talking to us from the intercom … the patient wanting to take some air. … it’s taking too long, but the amount of stress in her voice. We’re all outside waiting trying to reassure her, but we’re all stressed knowing that she’s alone … no one’s allowed in that room until everything is set up properly. You feel isolated, abandoned, alone, helpless. You can’t do anything until somebody comes in.

Another nurse remarked: “your training and learning is useless and you’re just watching your patients slowly deteriorate and eventually die.” They referred to a specific situation where the feeling of being “useless” was amplified because they had to call the family and inform them: “whatever I told you in the morning does not apply anymore cause your loved one passed away.” This helplessness in which there was nothing the nurse could do to prevent the patient’s death led to feelings of “sadness.” One nurse explained the impact of not permitting visitors is that patients “die alone” which is “pretty awful” and “not fair to these poor patients.” Another nurse indicated that “the saddest part is … [the nurse] who’s in that room is also mentally and emotionally drained because they’re the only person with that patient” when they are dying. They elaborated that it was “emotionally upsetting. You go home and feel so terrible that it is the way it is. It has to be. … It’s depressing … it’s sad to witness.” Another nurse explained having a special connection with a patient who ended up dying of COVID: “I feel really sad … I find myself in the corner crying. That's when I think about existential ideas. Like, why this patient?” Although there was a tremendous amount of fear, uncertainty, helplessness and sadness, nurses’ stories also revealed elements of resilience.

#### Resilience

Resilience emerged as an important element of the emotional experience in relation to how nurses thrived and adapted despite stress and adversity. The gravity of the experience was articulated by one nurse: “[it] made me more resilient … I kept reminding myself … it's going to be a historical moment. As hard as it is … you'll look back and value what you learned.” They stated that although they felt “empty emotionally … going through the uncertainty” generated “a shield, so we give up a little, but then we also get a little in terms of our strength … just toughens you and makes you stronger.” The idea of a shield suggests the resilience that one gained from this experience will serve as protection in future situations. They elaborated that “I'm able to think more quickly on my feet, adapt to stressful situations, and just work with the resources I have and provide good patient care. I do think it'll help me in future stressful situations.” As noted, “there's no resiliency without the struggle, without the adversity” and that COVID-19 “positively affected it … we get creative on how to support each other given these adversities.” One of the nurse leaders remarked that COVID-19 “challenged me to be resilient and adaptable to change. … every single day, there's a new policy coming out. There's new evidence.” They indicated: “I give credit to my staff, I see them being more resilient, understanding that change is something that's normal … we just have to take it in stride.” As we found, the myriad of emotions experienced were highly influential and had an element of agency upon nurses.

### The agency of emotions

Nurses’ stories revealed how emotions moved a person. The agency of emotions included powerful and persistent emotions, containing/releasing emotions, isolation, emotional and physical exhaustion, and emotional labour.

#### Powerful and persistent emotions

The agency of emotions included powerful and persistent emotions enduring tenaciously over time. One nurse commented that work and COVID is “always in the back of your mind … it messed with me mentally … at the end of SARS, I thought that was bad. And this is a lot worse.” It was explained that COVID was different because “we had to deal with it everyday.” It was common for nurses to be overcome with emotions during interviews: “Um [pause for four seconds], I mean, I’m so emotional … [choking up and crying] just remembering these things.” They explicitly likened the emotions to “post traumatic stress disorder [PTSD].” Another nurse described how it feels like it “will just blow up in my head. Deep inside me, I'm exploding because of … all the emotions that I'm accumulating from everybody.” The traumatic nature of these emotions are highlighted in this excerpt where a nurse described the impact on the nurse when family say good-bye to their loved one over “video” or “telephone”:

You just look at your colleague and ask them that you need five minutes to go cry in a hallway by yourself … we just keep chugging on, but I wonder what's going to be left if, if this goes on for longer periods of time. Left of us, I mean.

The traumatic nature of the emotions is underscored in this excerpt when the nurse indicated that they will lose part of themselves. Nurses noted taking “those situations home, it was hard to cope” watching family members say “goodbye … through a glass door … seeing it almost on a daily basis.” The intensity of the emotions were highlighted in nurses’ descriptions of reliving the trauma: “I couldn't erase the images out of my head, and I would replay some of those images that I saw.” This nurse elaborated “I found myself very sad, crying, very angry. I just felt there wasn't a light at the end of the tunnel.” It was common for nurses to indicate problems sleeping, one nurse described nightmares with “blood coming off the bed … I couldn't go back to sleep because I would feel overwhelmed.” They commented I was “losing [my] mind” and “my brain would race … I started losing it. I would be in the driveway just swearing like I f’n hate this. Like I don't want to do this anymore.” Although traumatic effects are already being seen, it was noted that many of the effects will not be seen until later: “I don't think the effects of what we're going through will be seen right away. I think it will be long term.”

#### Containing and releasing emotions

Nurses’ narratives revealed that they contained and released emotions in terms of holding them in and letting them go. One nurse said: “we weren’t able to have feelings until the end of the day” and further explained “don't even allow ourselves to have feelings, because how else would we cope and survive?” There was a nuanced reason for containing one’s emotions when in a leadership position: “I have not shared my emotions” because “I want to show that I'm a strong leader. I should not be showing weakness. If I do, my belief is that the people I lead would crumble.” At some point, the impact of containing one’s emotions led to a release: “we held it together on the unit, but … you’d go home, you tear up at the slightest thing. … I just release my stress through crying. … I cry things out in the shower or when I’m in bed.” Another nurse remarked how they found themselves “taking it from zero to 100 really quick.” Similarly, another nurse explained that at work I am “forced to put on a professional front … at home, I take off all that. I get really short tempered and annoyed.” The idea of putting on a front suggests that there are deeper underlying emotions. One nurse “I just felt angry and sad all the time … lashing out on people for things that I was bottling up.” Nurses were containing and releasing myriad of emotions.

#### Isolation

Isolation was an element associated with the emotional experiences. It was noted the “hardest part” was “isolating” from family. It was common for nurses to physically distance themselves from family engendering a feeling of isolation. One nurse described feeling “detached from my family” whereas another nurse noted feeling “alone … it was months before I saw anybody … that was very hard to deal with and took a big toll.” One nurse described the somber experience of visiting a family member on a momentous occasion outside a “window”:

She’s crying, trying to see how I was doing. That was probably my lowest day. I actually broke down crying … you have this exciting, new milestone in your family … that you can't even be a part of. So that day was definitely one of my lowest, that was really sad. And the drive to [city] and the drive back in the same day, that was a long day and to do that all alone, like sucked.

It was noted that “loneliness is a great part of the whole journey” of COVID in terms of “not being able to see the ones I love … quite anxiety producing, very lonely and made me very upset.” The physical distancing was compounded because “not only at work do you have to socially distance from colleagues, but then you come home, you're alone … very depressing.” Narratives reflected that nurses felt contaminated: “People didn’t want to visit us. People didn’t want to have contact with that, that scariness so we were isolated in our little bubble.” In addition, there was an element of isolation and divide among nurses in the context of limited PPE: “there was a bit of nurses against nurses” explaining that “there were a lot of digs about, if you use it [N-95] today, are the rest of us going to have it in a few months? Are you putting your life ahead of everyone else’s? … and making people feel guilty.”

#### Emotional and physical exhaustion

Working in these environments led to emotional and physical exhaustion in terms of being drained of energy and strength. It was common for nurses to describe the experience as “utterly exhausting” and “draining and stressful” in terms of having to don “your N-95, your hairnet, your face shield, your gown. Then taking it back off when you're done. It tires you out.” They elaborated that you are “sweating, you're hot, very, very uncomfortable.” The emotional exhaustion was highlighted in the context of caring for multiple deteriorating and dying patients: “you want at least one person that you're taking care of to survive” noting that “it's hard when you are defeated from the battle of trying to support yourself emotionally, and then having to provide that same or similar type of support to the family.” One nurse described feeling “stressed” and “very emotionally drained … definitely an emotional feeling of exhaustion.” The perpetual and recurring nature of the situation was unrelentingly emotional for nurses: “I was getting more and more tired … lots of nights [sniffling, pause for seven seconds] worried, like [sniffling, pause for five seconds] depending on the day at work, you’d sleep probably for two to three hours and come back to work [crying].” Another nurse noted: “we're always waiting for the next shoe to drop in terms of people coming through our door because we get the sickest … it's emotionally exhausting just because of all of that unknown.” They commented “getting battle weary … there's no end in sight, it eats away at your resilience … we are getting tired and that just wears on us.” Another nurse stated that “you’re physically exhausted and it takes a toll on your mental health because you’re just done, you’re just absolutely done.” The impact of this exhaustion led to coming home and wanting to “crawl into bed and not wake up until we have to go back to work” and “the next day I pretty much don't have energy as well.” The emotional and physical exhaustion was also related to the associated emotional labour.

#### Emotional labour

The agential nature of emotions involved emotional labour related to the work of emotional management and navigating one’s feelings. Nurses often talked about how their experience was “emotionally taxing” because of carrying emotions:

You're taking on the emotional load of people in the hospital, family members that can't see their family … you come home and are isolated … it took a stress, in that you couldn't turn it off. I was never not thinking about the pandemic.

A characteristic of emotional management involved controlling one’s emotions. One nurse described not wanting “to appear emotional” when working with families where their loved one was dying: “I had to make my way out of that situation, collect myself and be strong for the family.” Another nurse described feeling “useless” and dealing with their own “emotions of distress” when a patient died:

The fact that you're unable to express those emotions puts you in a place where you're carrying this load. … Your emotions are not as important as helping someone who might need the help and might survive. So the baggage is, not only are you carrying whatever you had, but also trying to bottle it in so that you can move onto the next person, and that keeps on building.

Although nurses grappled with their emotions, it was clear that struggles to cope with them were near insurmountable at times.

An additional impact of grappling with emotions in the context of a dying patient was the distancing required due to COVID. It was explained that not having family present was emotionally difficult and “overwhelming.” Other nurses commented on the emotional work involved in discussions about death over the telephone or Zoom with family: “how do you tell someone that within a few hours your loved one’s going to die? Trying to support someone emotionally is hard to do over the phone … don't know what their expressions are, how they're gonna react.” Another nurse described it as “so hard, taking care of someone not being able to let their family in. It was emotional doing those phone calls saying this person is dying.” The emotional work was intense for nurses when attempting to involve family in the dying process:

Hearing those types of cries, over a phone and not being able to console family members breaking, watching their hearts break, it takes a part of your soul. It's had a great emotional toll on me … it's so hard to tell a family member, sorry you can't come in. … It almost feels like a part of my soul, I gave to them. After those traumatic moments happen, you leave a little more empty.”

The re-telling of these stories was emotional for nurses. Another nurse described a situation in which a patient was dying, and the partner had to physically distance:

I held the baby monitor to the patient's ear, and she held a monitor from outside of the hallway. I just stood there and listened to her beg him to wake up. It was the hardest thing … she just broke down and at that point, COVID is out of our minds, we were just hugging each other and crying … that still has taken a big emotional toll on me. I can only imagine how traumatic that was for her.”

The intensity of the emotional labour that nurses experienced was powerful.

Emotional labour appeared in nuanced ways in nurse leaders’ narratives. Although the pandemic was emotionally difficult, one leader noted “I decided this is my dedication to nursing, to staff … I took that role of, I’m here to help. I’m here to support you.” There was also an element of emotional labour in maintaining confidential health accommodations. It was noted that nurses do not always recognize the “rules” that a leader has to follow: “many of them came into my office and yelled or cried saying I wasn't being fair, I was treating one person better than the other.” They described that it was the “hardest thing for me. I was emotional sitting in my office, astounded by what happened … yelled at for saying I wasn't doing my job properly.” The role of nurse leaders became arduous for some when grappling with the emotions of staff:

They share how fearful they are for their lives and their family and the potential of them bringing this COVID-19 virus home … imagine the magnitude of people sharing their thoughts and anxiety and stress about the pandemic … it's taking a toll on me and my psychological and physiological aspect of myself.

A deeper understanding of the emotional toll emerged in another leader’s interview who recognized the severity of emotions that staff were experiencing, but found it “hard” to “carry their emotional baggage” and almost be like a “counselor”: “It’s a lot of talking it out. … I feel like there should be somebody else who was a professional that's able to counsel somebody with PTSD. A lot of them are suffering, literally, from PTSD.” One leader described struggling because they were supporting staff, but “didn't have anybody to go to with what I felt, so I bottled it all up.” The severity and ripple effect of emotions was problematic as reflected in these narratives.

### How emotions shape nursing and practice

This element of agency intertwined with emotions also acted to shape nursing practice and nursing role identity and philosophy.

#### Nursing practice

The interconnected emotions acted to influence nursing practice in terms of generating more cautious and intentional practices. Nurses commonly remarked how the “fear” made them “more cautious” and “hypervigilant with hand hygiene, donning and doffing.” In terms of PPE, nurses spoke about monitoring self and each other: “those emotions were in a way helping me guide my practice … monitor myself in doing the proper techniques.” It was noted: “if one of us is forgetting a certain part of the donning or doffing process, we see that something's not being done as it should be, we kind of watch out for each other and we have remained vigilant.” They explained that it is “stressful” and “keeps you on edge … you wouldn't want to end up infecting yourself.” It was common for nurses to highlight the importance of following PPE procedures even in difficult situations:

I was worried this patient was going to decannulate themselves … but I was terrified at the idea of running into the room and not having the N-95 sealed … I did the right thing by making sure that I'm safe.

Fear of transmission also influenced how nurses organized their care: “everybody was scared. We didn’t want to go into [patients’ rooms]” so they talked about the importance of “cluster[ing] our care to minimize the amount of time we’re spending in the patient’s room … the longer I’m spending in this room, the risk of infection is higher.” The emotional experience also affected nurses’ assessment practices. They noted “monitoring” patients “very closely because I saw firsthand how fast that person deteriorated. And I would want to catch something early.” Another nurse commented on how their practice changed because of the trauma they felt when a patient deteriorated and died:

Noticing any changes and pinpointing that … having interventions such as putting them in isolation as soon as possible. In a way, the trauma, how it felt for me, how traumatic it was, was actually quite beneficial. It allowed me to realize how I could do better to prevent things from happening.

In addition to affecting their PPE and assessment practices, the emotional experience influenced nurses’ approach to patient engagement.

As a result of clustering care and less patient “check-ins”, nurses’ capacity for empathy and patient connection was influenced in two main ways. First, nurses found that they were at risk of becoming “task oriented … and that human interaction is being decreased.” Units were short staffed and staff were exhausted, which influenced their nursing practice negatively at times: “you kind of go in and it's ABC, just do what you have to do. You're taxed, you don't really have a lot, emotionally to give those patients that sometimes probably need it. I try my best. But there are times you're just tired.” The second main way that emotions affected their nursing practice was in highly positive ways in terms of inspiring them to be more “humanistic” and “connect with patients … on an emotional level.” As one nurse said “we were more into, let’s find out more about this person, what their story is … we had an iPAD … tried to connect patients to families.” It was noted that it made them more “empathetic as a nurse” and “more compassionate … you have to be in someone's shoes to really experience it.” One described “treating patients as if they were my own family” as well as providing: “therapeutic communication with these family members [via the telephone], because I can't even imagine what they go through not being able to come in and being able to be at the bedside.” The emotional impact on practice also challenged nurses to consider their nursing identity and philosophy.

#### Nursing role identity and philosophy

Emotions shaped how nurses thought about their identity and philosophy in terms of how they see themselves as a nurse and their key beliefs. Although they were fearful, it was clear that the pandemic reinforced nurses’ commitment to the profession: “people needed help” and “I felt an ethical requirement to pick up shifts.” This nurse elaborated how the situation made them feel in terms of their identity: “because of seeing their [Italian nurses] great sacrifice … I was like, wow, amazing, like I'm a nurse, they're a nurse.” Similarly, another nurse explained that “we’re all in this big giant COVID boat. … I felt more important than ever before.” The reinforcing of one’s identity was common. As one nurse said, they always “looked at nursing as part of a calling in my identity” and during COVID “you hope to bring help and comfort … you want to protect peoples’ lives.” Tensions typically emerged when the experience did not align or challenged their beliefs about nursing. In terms of limiting time in the patient’s room, nurses noted “I wasn't being the nurse that I usually am because I didn't want to put myself at risk” and how this “negates the whole essence of what nursing is. I was pretty sad about that.” They described how delaying care for a deteriorating patient while they appropriately donned PPE felt “wrong … [but] they told us at the beginning, you're going to have to go against all of your instincts and just think, did I put everything on properly?” The emotional experience of not having proper PPE based on the existing evidence also legitimately challenged nurses’ values:

Like how is this normal? How do we have to go somewhere and just pray that we're not going to die? … I have struggled with the idea of leaving nursing. And I never ever would have thought of that before this.

Nurses also referred to how these difficult “emotional experiences” prompted them to think about how they “connect with patients on a deeper level” as well as how “nursing is more than just keeping people alive. It's making the time in their lives count … kind of showed me a different way of how to nurse and how nursing should be.”

## Discussion

This study explored nurses’ emotional experiences of working in COVID-19 acute care hospital environments. The emotionally charged quality of nurses’ stories highlighted the intensity and complexity of their experiences, which unfolded in ways that revealed the trauma and loss nurses lived (and continue to live) through. Some of this trauma can be categorized as vicarious which is described as “profound psychological effects” that can disrupt a healthcare provider’s feelings of safety, power, and esteem, and last for a sustained period of time as a result of working with clients who have experienced trauma (McCann & Pearlman, 1990, p.133) – and COVID-19 has been traumatic for many. While loss was prominent, and an inevitable part of sacrifice, nurses also shared accounts of how their professional identity and practice were positively impacted, thus pointing to possibilities for gain to co-exist alongside loss. Similar to other COVID-19 research (Iheduru-Anderson, 2020; Sun et al., 2020), nurses in our study shared feelings of fear, isolation, anger, and frustration. They recounted how unrelenting fears of contracting the virus and infecting others drove them to sacrifice seeing family and friends. Echoing other research (Bagnasco et al., 2020; Han et al., 2020), the sense of emptiness and loneliness nurses experienced as a result of diminishing contact with loved ones compounded the existing emotional burden of working in COVID-19 environments.

A shortage of PPE and limited access to it on a daily basis aggravated their fear and apprehension even further, placing many into unfair situations in which they felt unsafe. Like many other nurses who have risked their safety during the pandemic (Maben and Bridges, 2020), anger and frustration emerged in their stories as a result of feeling inadequately protected. Not making nurses’ health and safety a priority sends a troubling message that “some lives appear to matter less” (Maben & Bridges, 2020, p. 2743). The emotional impact of these experiences left many in our study feeling depleted but witnessing patients suffering in isolation also inspired nurses to continue to engage in practice and approach care with increased empathy and compassion.

Fears of becoming infected challenged nurses’ ability to provide the kind of care they felt patients deserved, however, the perceived loss of connection reminded many of their professional and moral belief in providing compassionate care. Although some questioned leaving the profession due to emotional distress and lack of support, which was similar to other research (Labrague & de los Santos, 2020; Maben & Bridges, 2020), most nurses in our study felt that the emotional experience reinforced their commitment to nursing. Despite the intensity of the emotional distress, finding ways to enhance humanistic practice generated a sense of professional growth and strengthened their nursing identity. However, we cannot downplay how emotionally drained nurses felt as a result of the emotional labour required to provide compassionate care. Nurses often took on the weight of and prioritized patients’ and families’ emotions, which limited space to explore and express their own feelings. The nature of their emotional exhaustion is consistent with the compassion fatigue described elsewhere in terms of emotional experiences during the pandemic (Alharbi et al., 2020). The findings from our study as well as other COVID-19 research (Ruiz‐Fernández et al., 2020) highlight both the cost and reward of providing compassionate care, namely the feelings of exhaustion and fulfillment that can result from such commitment to care. In light of the intense demands nurses face as the pandemic continues, Schutz and Shattell (2020) emphasize the importance of supporting nurses’ psychological well-being to facilitate the release and reconciliation of negative emotions.

It was apparent that nurses still felt the intensity of the experience as they faced ongoing, pent-up emotional turmoil in reliving vivid, recurring memories of patients and families suffering. Like other nurses who have become intimately acquainted with patient suffering outside the COVID-19 context (Alharbi et al., 2020), the emotional impact of watching patients suffer was amplified when they felt that their care efforts were futile in relation to changing patient outcomes. Moral tensions also emerged when contact restrictions and risks to personal safety were at odds with providing timely, high quality care, which aligns with other researchers’ findings (Gujral et al., 2020). Nurses in our study felt traumatized by these experiences, which pushed their emotional limits and radically disrupted their moral beliefs surrounding the delivery of good patient care, particularly when patients died unaccompanied by loved ones. They experienced immense helplessness and sadness as a result of these morally distressing situations. The ethical implications of patients dying in isolation have become more apparent now than ever during the COVID-19 pandemic (Gallagher, 2020), not only for those in their final moments of life but also for nurses as our study showed. Although we have yet to fully realize the long-term impacts of these experiences, nurses in our study recognized that the infiltrative and persistent nature of their emotional distress was reflective of PTSD. Symptoms of PTSD have also been reported among other front-line nurses working during the COVID-19 pandemic in Wuhan, China (Tan et al., 2020). Additionally, lessons learned from the H1N1 pandemic suggest that ensuring access to mental health support and providing information about the virus in a timely manner are critical to mitigating psychological distress among providers, especially in care settings where risk of infection is increased (Stelnicki et al., 2020). While we cannot and should not understate the significant emotional toll nurses endured, Gallagher (2020) emphasizes that we need to turn our focus towards understanding these negative experiences in relation to how nurses’ resilience can be better supported.

Amidst emerging research, which largely focuses on the emotional hardship and negative experiences nurses faced during COVID-19 (Han et al., 2020; Shahrour & Dardas, 2020; Shreffler et al., 2020), our study provides an additional angle that includes positive elements of strength, learning, and resilience. Although the uncertainty of the situation and lack of a clear endpoint represented one of the toughest challenges for nurses, confronting these difficult experiences enabled them to realize their capacity to adapt to adversity. These accounts portrayed resilience, which Henshall et al. (2020) conceptualize as “the ability to cope successfully despite adverse circumstances” (p. 3597). They also demonstrated what Rushton (2018) refers to as moral resilience in that many remained strongly committed to providing compassionate care despite the pandemic imposing ethically challenging situations. Nurses’ stories of resilience revealed that loss does not always have to be the sole outcome of sacrifice. Although PTSD has been explored in relation to the trauma nurses experienced during COVID-19, nurses’ accounts of strength and learning also suggest the potential for post-traumatic growth, which Nishi et al. (2016) characterize as positive changes that follow the hardship of experiencing trauma. Supporting nurses’ resilience is necessary to counterbalance the loss and trauma nurses go through and recalibrate the balance of give and take in which nurses have already given so much.

## Implications

The main message from this research is to consider how to better support nurses in settings that are emotionally-laden such as the COVID-19 clinical environment. With supportive environments, organizations can reduce the impact of untreated trauma and distress and limit long-term impacts on nurses. A simple support is to ensure transparency and flow of information and access to PPE coupled with guidance for infection prevention and control for nurses in their own home spaces. At the peak of pandemics, we suggest that nurses would benefit from the support of: *relief nurses* who can cover a nurse for emotional-health breaks; and *emotional support persons* (e.g., a therapist) to be present during shifts to connect with nurses and help them deal with their emotions and work through the emotional labour. Although this requires financial investment, the long-term impact upon nurses and thus, the financial long-term impacts may be worth it. Exploratory and intervention research about how the above supports influence nurses’ emotional health would be advantageous to future pandemic preparations. Also, because of the inclination for many to assume that emotions are negative in these types of situations, it is important that researchers seek out positive emotions to understand how working in these environments can be rewarding.

## Conclusion

Healthcare leaders and government bodies need to make sure that a nurse’s sacrifice is not all-encompassing, but that nurses are supported so that there is something left of them at the end of it all. And in saying that, we should want more than just something left, we should want nurses to feel that although they struggled, they also flourished in the face of adversity. They gained despite the loss, and they came out the other side as strong, resilient, and whole despite the challenges and adversity.
